# The genome of a steinernematid-associated *Pseudomonas piscis* bacterium encodes the biosynthesis of insect toxins

**DOI:** 10.1099/acmi.0.000659.v3

**Published:** 2023-10-05

**Authors:** Ryan Musumba Awori, Prasad Hendre, Nelson O. Amugune

**Affiliations:** ^1^​ Elakistos Biosciences, P. O. Box 19301-00100, Nairobi, Kenya; ^2^​ International Centre for Research on Agroforestry, P. O. Box 30677-00100, Nairobi, Kenya; ^3^​ Department of Biology, University of Nairobi, P. O. Box 30197-00100, Nairobi, Kenya

**Keywords:** bacterial insect toxins, biosynthetic gene clusters, digital DNA-DNA hybridisation, nematode microbiota, orfamides, *Pseudomonas piscis*, *Xenorhabdus*

## Abstract

Several species of soil-dwelling *Steinernema* nematodes are used in the biocontrol of crop pests, due to their natural capacity to kill diverse lepidopteran species. Although this insect-killing trait is known to be augmented by the nematodes’ *Xenorhabdus* endosymbionts, the role of other steinernematid-associated bacterial genera in the nematode lifecycle remains unclear. This genomic study aimed to determine the potential of *Pseudomonas piscis* to contribute to the entomopathogenicity of its *Steinernema* host. Insect larvae were infected with three separate *Steinernema* cultures. From each of the three treatments, the prevalent bacteria in the haemocoel of cadavers, four days post-infection, were isolated. These three bacterial isolates were morphologically characterised. DNA was extracted from each of the three bacterial isolates and used for long-read genome sequencing and assembly. Assemblies were used to delineate species and identify genes that encode insect toxins, antimicrobials, and confer antibiotic resistance. We assembled three complete genomes. Through digital DNA–DNA hybridisation analyses, we ascertained that the haemocoels of insect cadavers previously infected with *Steinernema* sp. Kalro, *Steinernema* sp. 75, and *Steinernema* sp. 97 were dominated by *Xenorhabdus griffiniae* Kalro, *Pseudomonas piscis* 75, and *X. griffiniae* 97, respectively. *X. griffiniae* Kalro and *X. griffiniae* 97 formed a subspecies with other *X. griffiniae* symbionts of steinernematids from Kenya. *P. piscis* 75 phylogenetically clustered with pseudomonads that are characterised by high insecticidal activity. The *P. piscis* 75 genome encoded the production pathway of insect toxins such as orfamides and rhizoxins, antifungals such as pyrrolnitrin and pyoluteorin, and the broad-spectrum antimicrobial 2,4-diacetylphloroglucinol. The *P. piscis* 75 genome encoded resistance to over ten classes of antibiotics, including cationic lipopeptides. Steinernematid-associated *P. piscis* bacteria hence have the biosynthetic potential to contribute to nematode entomopathogenicity.

## Data summary

The accession numbers for complete genomes of *Xenorhabdus griffiniae* Kalro, *X. griffiniae* 97, and *Pseudomonas piscis* 75 are CP133479, CP133647, and CP133164, respectively. The raw reads used to assemble these genomes are available from the SRA database through accession numbers SRR25805238, SRR25809462, and SRR25793747. Raw data from various genome analyses are found in Supplementary Workbook 1.

## Introduction

The abundance of nematodes is exceeded by no other animal [1]. Yet only the steinernematids and heterorhabditids, which consist of the genera *Heterorhabditis, Neosteinernema,* and *Steinernema* [2], are classified as entomopathogenic [3]. *Steinernema* IJs are naturally found in soils the world over [4]. IJs seek out insect prey and gain entry into the haemocoel through either natural openings or by burrowing through the insect cuticle [5]. Once within the haemocoel, IJs release their bacterial symbionts which include *Xenorhabdus, Pseudomonas*, *Alcaligenes *, *Stenotrophomonas *, *Serratia, Ochrobactrum, Enterobacter, Pseudochrobactrum,* and *Brevundimonas* [6–11]. Upon detection of insect haemolymph, *Xenorhabdus* bacteria secrete [12] a pot pourri of natural products that ultimately contribute to both the fecundity and entomopathogenicity of their nematode host. For other bacterial endosymbionts, their specific role in the nematode lifecycle remains unclear. However, several strains have been demonstrated as entomopathogenic. For example, *Alcaligenes faecalis* AL618 and *Serratia marcescens* CAST5, which were isolated from the haemocoel of *Steinernema feltiae*-infected *Galleria mellonella* cadavers, were entomopathogenic to both lepidopterans and dipterans [10]. *A. faecalis* MOR02, which was associated with both *S. feltiae* and *S. carpocapsae* IJs, was entomopathogenic to *G. mellonella* [8]. Strains of *Pseudomonas protegens* and *P. chlororaphis* that were endosymbionts of *S. carpocapsae, S. glaseri,* and *S. weiseri,* were highly entomopathogenic to *Spodoptera littoralis* [7]. Moreover, these *P. chlororaphis* and *P. protegens* strains were resistant to antimicrobials produced by bacteria with whom they shared a nematode host [7]. *P. protegens* C01 from *S. feltiae* was entomopathogenic to both lepidopterans and dipterans via both oral and intrahaemocoel routes [11]. The *P. protegens* C01 genome contained *fitD*, a robust marker of entomopathogenicity in a pseudomonad [13] since it is part of a seemingly non-cryptic BGC that encodes the production of the Fit toxin [14]. Other insect toxins produced by entomopathogenic strains of *Pseudomonas* include orfamide and rhizoxin [15]. This predicted and experimentally validated entomopathogenicity of pseudomonads led to the hypothesis that they contribute to the entomopathogenicity of steinernematids [16]. Since the production of *Pseudomonas* natural products such as insect toxins is strain-specific [13], the isolation of more *Steinernema*-associated strains that either encode or secrete insect toxins would further support this hypothesis.

Three EPNs isolated from soils in Kenya *– Steinernema* sp. 97, *Steinernema* sp. Kalro, and *Steinernema* sp. 75 – were entomopathogenic to *Tuta absoluta* [17, 18]. In this study, we isolated three bacteria from insect cadavers infected with *Steinernema* sp. 97, *Steinernema* sp. Kalro, and *Steinernema* sp. 75. For these bacterial isolates, we characterised their genomes, delineated their species, and conducted genome analyses to determine the potential of the non-* Xenorhabdus* bacterium to contribute to the entomopathogenicity of its nematode host. Specifically, we identified and analysed genes encoding not only secondary metabolites, including insect toxins, but also the resistome in a *Steinernema*-associated strain of *Pseudomonas piscis *. Our findings demonstrate that *Steinernema*-associated *Pseudomonas piscis* have the potential to produce insect toxins and subsequently dominate the insect cadaver through both antimicrobial production and resistance.

## Methods

### Isolation and morphological characterisation of nematode-associated bacteria

Three nematode cultures that had been previously been isolated, characterised, and reposited at the EPN nematode collection of the Horticulture Research Institute, KALRO, Thika, Kenya, were used in this study: *Steinernema* sp. 75, *Steinernema* sp. Kalro, and *Steinernema* sp. 97, which originated from soils in the Rift Valley, Thika, and Central regions of Kenya, respectively [17]. Bacteria were indirectly isolated from each of the three, through the haemolymph of *G. mellonella* larvae as previously described [19]. Briefly, 8–12 larvae were infected with a nematode culture for each treatment. Treatments were monitored for insect death and subsequent nematode emergence from at least one cadaver. This happened four days post-infection for *Steinernema* sp. Kalro treatments and seven days post-infection for both *Steinernema* sp. 75 and *Steinernema* sp. 97 treatments. Limp cadavers that were void of putrefaction, were then selected for bacterial isolation. They were surface-sterilised and dissected to obtain internal haemolymph/fluid of degraded tissues. This was then streaked onto LB [20] agar plates (1 % tryptone, 0.5 % NaCl, 0.5 % yeast extract, 1.2 % agar [w/v]), which were incubated at 28°C for 3 days. To determine ampicillin resistance, pure cultures were plated on LB agar supplemented with 50 and 100 µg ml^−1^ ampicillin and incubated at 28 °C for 3 days. Pure cultures were similarly grown on nutrient agar plates. Catalase production was determined by mixing a single colony with 30 % (v/v) hydrogen peroxide (10 µl). Production of bubbles indicated the bacterium strain as catalase-positive.

### Bacterial DNA extraction

Pure plate cultures of the three isolates were used for DNA extraction with a Zymo Quick DNA Miniprep Plus extraction kit (Zymo Research, Irvine, USA), as per the manufacturer’s instructions. The concentration of DNA samples was measured with a Qubit 2.0 fluorometer (Thermofisher Scientific, USA) as per the manufacturer’s instructions for broad-range settings. DNA samples of *X. griffiniae* Kalro, *P. piscis* 75, and *X. griffiniae* 97 had concentrations of 967 µg ml^−1^, 155 µg ml^−1^, and 242 µg ml^−1^, respectively. These were then stored at −20 °C, until shipment to Plasmidsaurus (Oregon, USA) for genome sequencing.

### Genome sequencing

Libraries were first created using Oxford Nanopore Q20 +v0.14 library chemistry kits. Samples were multiplexed. Primer-free long-read sequencing was then conducted on an ONP platform employing R 10.4.1 flow cells. Bases were called with Bonito v 3.5.2 using a high-accuracy base call model – dna_r10.4.1_e8.2_260bps_sup@v3.5.2. Reads were demultiplexed with Guppy using a 70 % threshold. Individual read datasets were obtained in the fastq format. For the *P. piscis* 75 sample, the raw read set contained 901729912 bases from a single sequencing run that lasted 2 h 26 min. For the *X. griffiniae* Kalro sample, the cumulative raw read set contained 319129049 bases from two sequencing runs that individually lasted 9 h 20 min and 5 h 13 min. For the *X. griffiniae* 97 sample, the cumulative raw read set contained 235609660 bases from two sequencing runs that individually lasted 9 h 20 min and 4 h 50 min.

### Genome assembly

Raw read sets were used to assemble genomes with Trycycler [21] on the Galaxy EU webserver [22]. To assemble the P. *piscis* 75 genome, the same raw read set was first used to create separate assemblies in Flye [23], Canu [24], and Raven [25], using the default parameters of ONP reads. Raw reads were filtered in Filtlong by culling the worst 5 % of reads. This dataset was used to create both an assembly in Flye and 12 subsamples using the Trycycler subsample programme. Three, two, and three subsamples were used to create separate assemblies in Flye, Canu, and Raven, respectively, using default parameters for ONP reads. The twelve resultant assemblies were then used as input data for the Trycycler cluster programme. Assemblies that did not form clear clusters were culled and the remaining 11 assemblies were re-run through Trycycler cluster. Eleven contigs that formed a clear cluster, which represented the bacterial chromosome, were used in Trycycler reconcile/msa programme to not only circularise the sequences but also create an MSA. Using the Trycycler partition programme, the specific reads that were used to create contigs from this cluster were partitioned from the main set of reads. This partitioned set of reads, the MSA, and circularised contigs were used as input data in the Trycycler consensus programme to create a complete genome assembly. A similar workflow was used to assemble genomes of *X. griffiniae* Kalro and *X. griffiniae* 97. For these two, the final assemblies were additionally polished with Medaka [26]. All three assemblies were deposited in NCBI Genbank as complete genomes.

### Genome analyses for species delineation, and prediction of secondary metabolites and the resistome

To determine the characteristics of genome assemblies and their suitability for downstream genome analyses, genome assemblies were first analysed with the comprehensive genome analysis and annotation tools, on the BV-BRC platform [27]. Species delineation was conducted on TYGS [28] as previously described [19]. Briefly, genomes in fasta format were uploaded to the server for unrestricted analyses. Sequences of *16S rDNA* were extracted from the genomes. These were used to identify type strains that are most closely related to query genomes. Genome Distance blast Phylogeny (GDBP) distances were calculated between each of the query genomes and their mostly closely related type strains that have publicly available genomes. For the phylogenomic reconstruction, a distance tree was reconstructed using distances calculated from GDBP distance formula d5. The following genomes of non-type strains that were most closely related to the three query genomes were included in the dDDH analyses on the TYGS: *X griffiniae* XN45 (GCA_014656825.1), *X. griffiniae* VH1 (GCA_015163655.1), and *Pseudomonas* sp. CMR5c (GCA_003850545.1). The resultant phylogenomic tree from the analyses of pseudomonad strains was viewed in ITOL [29]. A scalable vector graphic of the tree was edited in Inkscape [30].

To identify BGCs that likely encode the production of secondary metabolites, genomes were uploaded to antiSMASH [31] and analysed under ‘relaxed’ strictness with the following analyses: known Clusterblast, Clusterblast, MIBiG cluster comparison, active sitefinder, RREFinder, cluster Pfam analysis, Pfam-based GO term annotation, TIGRFam analysis, and TBFS analysis. The modules and domains of predicted non-ribosomal peptide synthetases were further analysed to predict the amino acid building blocks and sequences of non-ribosomal peptides. A targeted search for the *fitD* gene was conducted. This is because genomes of close phylogenetic relatives of *P. piscis* 75 encoded the Fit toxin, which contributed to their entomopathogenicity [13]. Moreover, the Fit toxin is homologous to the make caterpillar floppy (mcf) insect toxin that is encoded in genomes of *Xenorhabdus* [32]. To identify the *fitD* gene, the nucleotide sequences of *Pseudomonas* genomes were queried with primer sequences TGGCTTTTATGTCCAAGGAC and TGGTTGGCGAAGTACTGCTC [32] in Geneious [33]. For identification of the *hcnA-C* BGC, the annotation track of the genome was likewise queried with*‘hcnA’*. Known chemical structures of predicted biosynthesized compounds were obtained from the Natural Product Atlas [34], modified in the online applet of ChemDraw and exported images were edited in Inkscape. Antibiotic resistance genes were identified with ABRicate coupled to the CARD database [35] under default parameters on Galaxy [22]. Genomic loci of genes associated with polymyxin resistance were identified within the *P. piscis* 75 genome in Geneious [33]. To identify resistance-conferring mutations in *pmrAB,* orthologs from the *P. aeruginosa* ^T^ genome (GCA_AE004091) were used for protein-protein pairwise alignments in both Geneious and BLASTp [36].

## Results and discussion

### Morphologically-distinct bacteria predominate the haemocoel of cadavers, previously infected with *Steinernema* spp. Kalro, 97 and 75

We aimed to obtain *Xenorhabdus* strains, by indirectly isolating putative *Steinernema*-associated bacteria from the haemocoel of EPN-infected cadavers. Two morphologically-distinct bacteria were the dominant culturable bacteria in *G. mellonella* cadavers infected with *Steinernema* spp. Kalro, 97, and 75 (Table 1, Fig. S1, available in the online version of this article).

**Table 1.** Morphological and biochemical characteristics of steinernematid-associated bacterium strains

Bacterium strain	Strain 75	Strain Kalro	Strain 97
Nematode host	*Steinernema* sp. 75	*Steinernema* sp. Kalro	*Steinernema* sp. 97
Pigmentation on Nutrient Agar	White	Rustic brown	Rustic brown
Elevation	Flat	Umbonate	Umbonate
Form	Regular	Irregular	Irregular
Margins	Entire	Entire	Entire
Texture	Mucoid	Mucoid	Mucoid
Swarming motility on 1.2 % agar plates	^+^	^+^	^+^
Catalase production	^+^	−	−
Ampicillin resistance (100 µg ml^−1^)	^+^	−	−
Ampicillin resistance (50 µg ml^−1^)	^+^	−	−

(+) Positive, (−) Negative.

Specifically, four days after infection of larvae with *Steinernema* sp. Kalro, the dominant cultivable bacteria from the haemocoel of cadavers had the characteristics listed in Table 1. These same colonies were predominant in the haemocoel of *Steinernema* sp. 97-infected cadavers, seven days post-infection (Table 1). This difference in post-infection duration before the isolation of bacteria from the haemocoel was due to the different durations until IJ emergence. Differences in the number of EPNs that infected larvae cannot be excluded as a reason for the faster emergence *Steinernema* sp. Kalro IJs, as the number of EPNs in each inoculum was not determined. For *Steinernema* sp. 75-infected cadavers, the dominant cultivable bacteria in the haemocoel, seven days post-infection, had characteristics that differed from those of other treatments (Table 1, Fig. S1).

We hypothesised that all three of these bacterial strains are likely members of *Proteobacteria *. This is because *Proteobacteria* are typically the dominant cultivable coloniser of *Steinernema*-infected insect larvae, following insect death. For example, 48 h post-infection, the dominant cultivable bacteria in the haemocoel of *G. mellonella* cadavers that had been infected with *S. riobrave* 355, *S. riobrave* Oscar, *S. feltiae*, *S. carpocapsae* Kapow, and *S. carpocapsae* 25 were *Xenorhabdus *, *Pseudomonas *, *Serratia* and *Salmonella *, *Xenorhabdus* and *Pseudomonas *, and *X. nematophila,* respectively [6]. Similarly, at 72 h post-infection with *S. feltiae* CO1, *P. protegens* was the dominant bacteria in haemolymph from *G. mellonella* cadavers [11]. Relatedly, in *S. carpocapsae*-infected *Manduca sexta* cadavers, *X. nematophila* AN1 was the dominant bacteria in the haemolymph 48 h post-infection [37].

Among these *Proteobacteria,* the *Xenorhabdus* genus has characteristics similar to those of strains Kalro and 97 that are listed in Table 1 [38, 39]. However, pigmentation [39] and ampicillin resistance are species-specific traits. For example, *X. budapestensis* [40], *X. bovienii* [41], and *X. nematophila* are ampicillin-resistant, whereas *X. griffiniae* is not [42]. Our results support the hypothesis that, from four days post-infection, the predominant cultivable bacteria in the haemolymph of cadavers infected with either *Steinernema* sp. Kalro or *Steinernema* sp. 97 were ampicillin-sensitive strains of *Xenorhabdus,* while those in cadavers previously infected with *Steinernema* sp. 75 were of a different genus. *Steinernema* sp. Kalro and *Steinernema* sp. 75 are conspecific strains [17]. However, they had morphologically different bacteria that predominated haemocoels of larvae they had previously infected (Table 1, Fig. S1), at four and seven days post-infection, respectively. This difference in the predominant bacterial coloniser is unlikely due to duration post-infection, since temporal changes in the predominant cultures in *Steinernema*-infected cadavers were only observed up until 48 h [6]. It is likely due to differences in the geographical region of isolation of the nematodes. For example, *A. faecalis *, *S. marcescens *, and *P. protogens* were the prevalent bacteria in *G.mellonella* cadavers that had been infected with *S. feltiae* ALG18, *S. feltiae* CAST5, and *S. feltiae* C01, respectively. These three conspecific nematodes were isolated from geographically-distinct soils [10, 43]. Similarly, the haemocoel of dead *Tenbrio molitor* larvae previously infected with the exact same *Steinernema* species but then reared in geographically-distinct soils had significantly different bacterial communities, 10 days post-infection [44]. Taken together, we find support for the hypothesis that the haemocoel of *G. mellonella* larvae previously infected with either *Steinernema* sp. Kalro or *Steinernema* sp. 75, which are conspecific nematodes from geographically different soils, were dominated by different cultivable bacterial genera, from four days post-infection.

### Strains of *X. griffiniae* and *P. piscis* are isolated from *G. mellonella* cadavers previously infected with *Steinernema* nematodes.

To conclusively identify prokaryotic species, we sequenced and assembled the genomes of the three bacterial isolates (Table 1). We then assessed the quality of genome assemblies to determine their suitability for subsequent bioinformatic analyses. All three genomes had values for N_50_, completion, and contamination that were >4.5 Mb, >99.5 %, and <1.7 %, respectively (Table 2), making them suitable for species delineation through dDDH analysis. Bacterial strains Kalro and 97 were both delineated as *X. griffiniae* while bacterial strain 75 was delineated as *Pseudomonas piscis *. This is because their pairwise dDDH values with corresponding type strains were all above the 70 % threshold value for conspecific strains [45, 46]. Specifically, the pairwise dDDH values between corresponding type strains and strains Kalro, 97 and 75 were 70.3, 70.6, and 83.0 %, respectively (Supplementary workbook 1).

**Table 2.** Characteristics of genomes assembled in this study as determined on the BV-BRC platform [27]. Genomes were annotated with RAST-K [79]

	* X. griffiniae* 97	* X. griffiniae* Kalro	* P. piscis* 75
Genome length	4 559 032 bp	4 559 030 bp	6 752 883 bp
Contig N_50_	4 559 032 bp	4 559 030 bp	6 752 883 bp
G+C content	43.84%	43.84%	63.61 %
Assembly coverage	48 ×	65 ×	125 ×
Completeness	100 %	100 %	99.5 %
Contamination	0.2 %	0.3 %	1.7 %
Coarse consistency	99.2 %	99.2 %	98.7 %
Fine consistency	98.4 %	98.2 %	95.9 %
Total gene features	5074	5077	6475
Total protein-encoding genes	4438	4441	6306
Partial protein-encoding genes	0	0	0
Protein-encoding genes with functional assignments	3179 (71.6 %)	3176 (71.5 %)	4876 (77.3 %)
Protein-encoding genes without functional assignments	1259	1265	1430
Repeat regions	476	476	58
tRNA	81	81	71
rRNA	22	22	16

Bp, base pairs; CDS, coding sequences; tRNA, transfer ribonucleic acid DNA sequences, rRNA, ribosomal ribonucleic acid DNA sequences; G+C content, guanine + cytosine content.

**Fig. 1.**
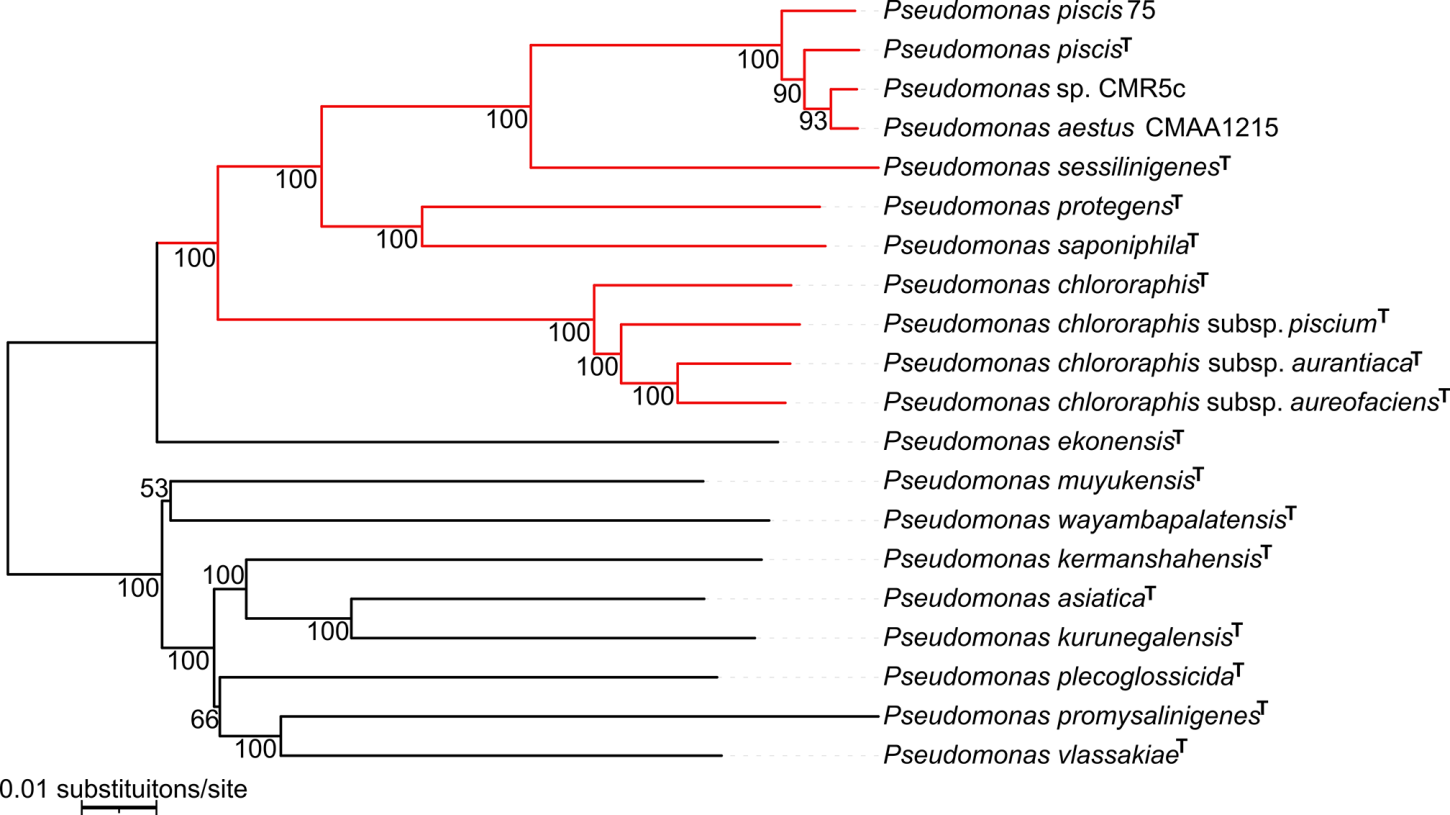
Neighbour-joining phylogenomic reconstruction of strains most closely related to *Pseudomonas piscis* 75 and *Pseudomonas* sp. CMR5c, as reconstructed with FASTME, using Genome Distance blast Phylogeny (GDBP) distances calculated from distance formula *d5* on the Type Strain Genome Server [28]. Strain 75 was isolated in this study and clustered in the *P. piscis* clade. Other members of *P. piscis* clade, strains CMAA1215 and CMR5c were previously demonstrated to both be conspecific with *P. piscis* [45, 80]. *P. piscis* strains fell within a clade containing both *P. protegens* and *P. chlororaphis* subgroups (red). This clade contains all insecticidal pseudomonads [13, 55]. Pseudo-bootstrap values from 100 replications are shown at nodes.

The bacterium isolates were hence renamed as follows: strain Kalro= *X. griffiniae* Kalro, strain 97= *X. griffiniae* 97, and strain 75= *P. piscis* 75. The difference in GC content between genomes of *X. griffiniae* Kalro and 97 was less than 1 % (Table 2), further validating them as conspecific strains [46]. *Xenorhabdus* are natural gut endosymbionts of *Steinernema* IJs [39] while strains from the *P. chlororaphis* clade, a sister clade to that of *P. piscis* 75 (Fig. 1), are frequently associated with *Steinernema* IJs [7, 16, 44]. Altogether, we found support for the hypothesis that *X. griffiniae* 97, *X. griffiniae* Kalro, and *P. piscis* 75 originated from the *Steinernema* IJs that infected the *G. mellonella* larvae.

**Fig. 2.**
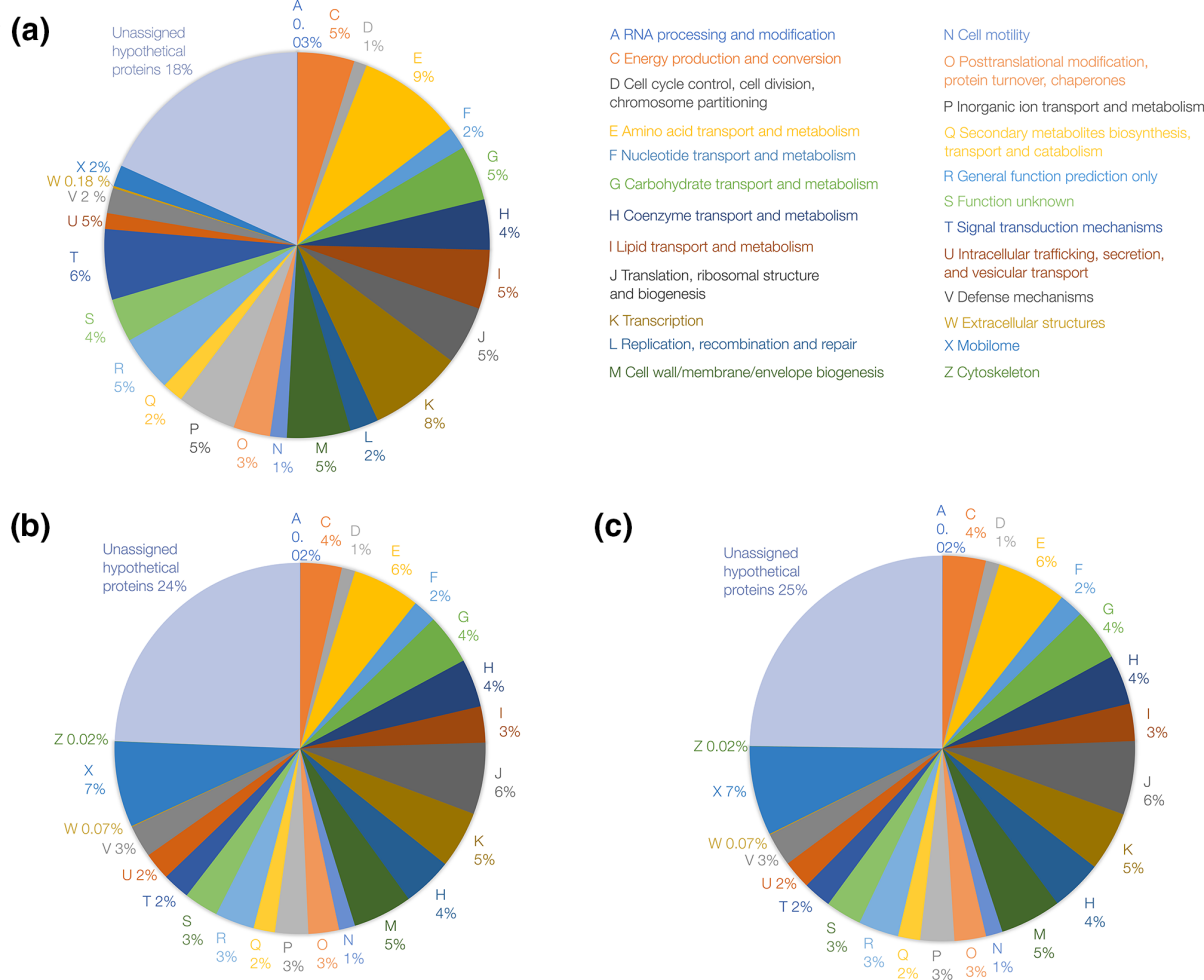
Pie charts representing the proportions of the proteome that are assigned to various functions in three steinernematid-associated bacteria. (**a**) *Pseudomonas piscis* 75 (b) *Xenorhabdus griffiniae* Kalro (c) *X. griffiniae* 97. Functional categories are from the NCBI COG 2020 database. The proportion of proteins with known functional assignments (this excludes categories R and S) for strains 75, Kalro and 97 were 73, 70 and 69 %, respectively.

From the RAST-K annotation analyses (Table 2), the proportions of protein-encoding genes with functional assignments in strains Kalro, 97 and 75 were 71.6, 71.5, 77.3 %, respectively. This was consistent with the proportion of protein-encoding genes with functional assignments from NCBI COG annotation analyses (Fig. 2). For both annotation analyses, the proportion was higher in the *Pseudomonas* than *Xenorhabdus* genomes. A likely reason for this higher proportion is the bias towards elucidation of gene functions in pseudomonads than in other prokaryotic taxa [47]. Despite their smaller size, both *X. griffiniae* genomes had 476 repeat regions compared to 58 in *P. piscis* 75 (Table 2). The *X. nematophila* ATTC19061 genome also had a considerably high proportion of repeat regions [48], suggesting that this may be a genus-related genome characteristic.

*X. griffiniae* endosymbionts of different *Steinernema* species from soils in Kenya belonged to one subspecies. This is because *X. griffiniae* strains Kalro, 97, VH1, and XN45 had pairwise dDDH values amongst them that all exceeded (Supplementary workbook 1) the 80 % threshold for subspecies [49]. We previously isolated *X. griffiniae* XN45 and *X. griffiniae* VH1 from *Steinernema* sp. scarpo and *Steinernema* sp. VH1, respectively, These nematodes were isolated from soils in Murang’a and Vihiga, Kenya, respectively [19, 50]. *X. griffiniae* Kalro and *X. griffiniae* 97, were isolated from *Steinernema* sp. Kalro and 75, whose natural habitats were soils in Thika and Rift Valley regions of Kenya, respectively [17]. Moreover, *Steinernema* sp. scarpo, *Steinernema* sp. Kalro and *Steinernema* sp. 97 are most likely three new species, as they each had at least 2.3 % *ITS rDNA* sequence dissimilarities [17, 51], with any described species. This sequence dissimilarity threshold often delineates closely-related *Steinernema* species [52, 53].

Although *Steinernema* sp. 75 and *Steinernema* sp. Kalro were conspecific, they differed in levels of entomopathogenicity – i.e the mean larval mortality rate in *Tuta absoluta* at 48 h post-infection with 150 EPN IJs – and geographic regions of isolation [17]. Our findings demonstrate that they also differed in the prevalent bacteria, *P. pisics* 75 and *X. griffiniae* Kalro respectively, found in the haemocoel of *G. mellonella* they had previously infected, four days post-infection. The soil habitat of *Steinernema* IJs were hypothesised as the likely source of its *Pseudomonas* microbiota [7]. These bacteria were then suggested to be vertically transmitted between nematodes [7]. Since the insecticidal repertoire of a pseudomonad is strain-specific [13, 54] we hypothesize that soils influenced the association of *P. pisics* 75 with *Steinernema* sp. 75, which in turn influenced nematode entomopathogenicity, resulting in conspecific nematodes from geographically-distinct soils, having different levels of entomopathogenicity.

### The genome of *P. piscis* 75 does not encode the Fit toxin but encodes other insect toxins and antimicrobials

*P. piscis* 75 belonged to the *P. protogens* subgroup (Fig. 1) [55], whose members were previously demonstrated to be highly insecticidal partly due to the Fit entomotoxin [13, 54]. We therefore analysed the *P. piscis* 75 genome to identify *fitD* [14] and other genes that encode the production of entomotoxic secondary metabolites.

Using previously designed primer sequences for *fitD* [32] as probes, we searched the four *P. piscis* genomes for *fitD*, which was detected in all but the *P. piscis* 75 genome. In strain CMAA1215, *fitD* (locus tag: P308_15705) was 8646 bp, and encoded 2682 aa, whereas in both CMR5c and *P. piscis* ^T^, *fitD* was 8991 bp and encoded 2997 aa (locus tags: C4K40_3850 and GDH07_25415, respectively). To verify the absence of *fitD* in strain 75, we used both *fitD* sequences to query the *P. piscis* 75 genome using blast [36]. Neither yielded the identification of an ortholog. Lastly, we identified and analysed all genes in the *P. piscis* 75 genome that encoded proteins between 1837–3714 aa (Supplementary workbook 1) because, in other *P. piscis* strains, *fitD* encodes a protein of either 2682 or 2997 aa. None of the identified genes were orthologous to *fitD*.

Genomes of 37/39 strains from the clade comprising both *P. protogens* and *P. chlororaphis* subgroups contained *fitD* [13]. The two strains that did not, *P. chlororaphis* ^T^ and *P. chlororaphis* subsp. *aurantiaca* ^T^, were associated with neither plants nor arthropods, unlike all strains that had *fitD*. These findings support the hypothesis that *P. piscis* 75 is a nematode-associated member of the *P. protogens* subgroup, whose genome does not contain *fitD*.

**Fig. 3.**
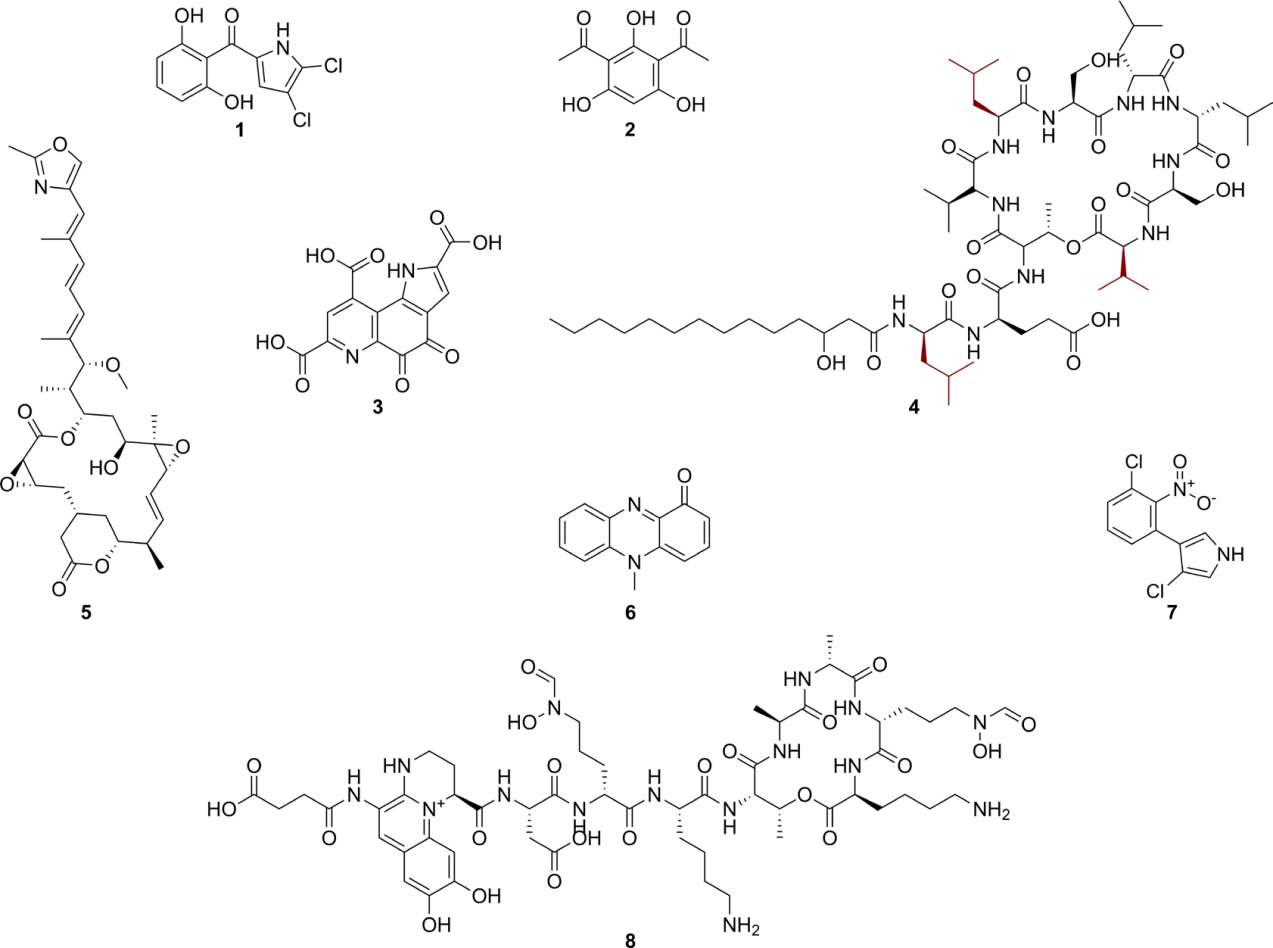
Known natural products whose production is predicted to be encoded by biosynthetic gene clusters (BGCs) found in the *Pseudomonas piscis* 75 genome.1, pyoluteorin; 2, 2,4-diacetylphloroglucinol; 3, pyrroloquinoline quinone; 4, novel orfamide B derivative; 5, rhizoxin; 6, pyocyanin; 7, pyrrolnitrin; 8, ferric-pyoverdine. The *orfABC* BGC was predicted to encode the synthesis of 4, which differs from orfamide B at the three building blocks highlighted in red.

The *P. piscis* 75 genome had 18 BGCs, nine of which were predicted to encode the production of known molecules with a variety of biological activities (Table 3, Fig. 3).

**Table 3.** Biosynthetic gene clusters (BGCs) in the *Pseudomonas piscis* 75 genome

	BGC	Type	Biosynthesis it encodes	Putative bioactivity	Locus
Known BGCs					
1.	*prnA-D*	Pyrrole	Pyrrolnitrin	Antifungal	4,127,239–4 132 801
2.	*phzA-H,M,S*	Phenazine	Pyocyanin	Cytotoxin	3,342,609–3 336 401
3.	*rzxA-I*	PKS	Rhizoxin A	Insect toxin, antifungal, antimitotic	3,346,472–3 424 870
4.	*pltA-R*	PKS-NRPS	Pyoluteorin	Antibacterial, antifungal	2,951,699–2 981 670
5.	*phlA-D*	PKS	2,4-diacetyl-phloroglucinol	Antibacterial, antifungal, antiviral, antiprotozoal, antihelminth, antimitotic	6,441,826–6 450 040
6.	*ofaA-C*	NRPS	Orfamide B	Insect toxin, antibacterial	2,351,633–2 386 072
7.	*hcnABC*		Hydrogen cyanide	biocide, insect toxin	2,859,952–2 862 912
8.	*pqqA-F*	Redox cofactor	Pyrroloquinoline quinone	Antioxidant	6,144,469–6 151 272
9.	*pvdIJDL*	NRPS	Ferric-pyoverdine	Siderophore	4,603,253–4,572,268 and 4,684,060–4,697,091
Unknown BGCs*					
10.		NRPS-PKS	^D^Asp containing NRP-PK hybrid		4,492,684–4 496 951
11.		ß-lactone			4,422,992–4 432 121
12.		Homo-serine lactone			5,480,867–5 491 545
13.		Aryl polyene			535,484–521 163
14.		Aryl polyene			5,068,530–5 082 729
15.		Terpene			5,266,698–5 284 670
16.		NAGGN			4,866,653–4 875 283
17.		RIPP-like			2,644,416–2 640 621
18.		CDPS			1,536,225–1 540 602

*Unknown BGCs are defined as those that encode the biosynthesis of lead molecules whose chemical structures remain to be elucidated.

CDPS, cyclic dipeptide synthase; NAGGN, N-acetylglutaminylglutamine amide; NRPS, non-ribosomal peptide synthetase; PKS, polyketide synthase; RIPP, ribosomally synthesised and post-translationally modified peptide.

The nine unknown BGCs were predicted to encode the production of uncharacterized molecules including a β-lactone, a homoserine lactone, a RIPP, a terpene, two arylpolyenes, and a ^D^Asp-containing non-ribosomal peptide-polyketide hybrid (NRP-PKS) (Table 3).

Among the known BGCs, *prnABCD* [56], *phzABCDEFGH* [57], *pltABCDEFGHIJKLMNOPQR* [58], and *phlABCD* [59] were predicted to encode the biosynthesis of the antifungal pyrrolnitrin, cytotoxin pyocyanin, antifungal and antibacterial pyoluteorin, and antimicrobial and antihelminth 2,4-diacetylphloroglucinol, respectively, while the *pqqABCDEF* BGC [60] was predicted to encode the production of the antioxidant pyrroloquinoline quinone. Three known BGCs were predicted to encode the production of compounds with insecticidal activity: *hcnABC* [61]*, ofaABC* [62, 63], and *rzxABCDEFGHI* [64, 65] encoded the production of hydrogen cyanide, rhizoxins, and orfamides, respectively (Fig. 3). The *pvd* BGC was predicted to encode the production of ferric-pyoverdines [66]. Pyoverdines are siderophores that are yellow and therefore are the possible cause for the tint of yellow observed in colonies of *P. piscis* 75 (Fig. S1). The *P. piscis* 75 *pvd* BGC encoded the production of an acylated octapeptide metallophore with the following linearised peptide backbone: Asp-^D^Fo-OH-Orn-Lys-Thr-Ala-^D^Ala-^D^Fo-OH-Orn-Lys, which is identical to those of ferric-pyoverdines from *P. protogens* CHA01 and *P. protogens* Pf-5 [66]. This agrees with the finding that all members of the *P. protogens* subgroup (SG) encode the production of pyoverdines [55]. It further suggests that members of the *P. protogens* SG biosynthesize pyoverdines that have an identical peptide backbone.

Orfamides are lipopeptides produced by the OfaA, OfaB, and OfaC non-ribosomal peptide synthetases. In *P. piscis* 75, the predicted OfaA protein contained a C_starter_ domain, which catalyses *N-*terminal acylation [67] that results in lipopeptides. The encoded OfaA, OfaB, and OfaC non-ribosomal synthetases were predicted to contain ten modules. We analysed these predicted ten modules, and using the Stachelhaus codes of the adenylation domains [68] and positions of dual epimerisation/condensation domains [67], predicted that they biosynthesize a peptide backbone of ^D^Leu-^D^Glu-^D^aThr-^D^Val-^D^Leu-^D^Ser-^L^Leu-^L^Leu-^D^Ser-^D^Val. This was most similar to the Orfamide B backbone but differed in the configurations of amino acids at position one (^L^Leu), five (^L^Leu), and ten (^L^Val) [63], suggesting that *P. pisics* 75 encodes the production of novel Orfamide B derivatives (Fig. 3). Orfamide B is not only both an antibacterial and insecticidal compound but is also the main cyclic lipopeptide biosynthesized by the CMR5c strain of *P. piscis* [63]. Orfamides were demonstrated to contribute to the oral entomopathogenicity of pseudomonads to *Plutella xylostella* [15].

The *hcnABC* BGC in the *P. pisics* 75 was predicted to encode the biosynthesis hydrogen cyanide, a biocidal compound produced by pseudomonads in both *P. chlororaphis* and *P. protegens* subgroups [13, 55]. In *P. protogens* ^T^, hydrogen cyanide was shown to significantly contribute to its entomopathogenicity via intrahaemocoel and oral routes to *G. mellonella* and *P. xylostella*, respectively [15]. The *rhiABCDEFGHI* BGC in the *P. piscis* 75 genome was predicted to encode the production of rhizoxins. These are broadly toxic macrolides that contributed to the entomopathogenicity of *P. protegens* Pf-5 to *Drosophila melanogaster* [64]. Taken together, these demonstrate that *P. piscis* 75 encodes the production of the insect toxins rhizoxins and orfamides, and the antimicrobials pyrrolnitrin, pyocyanin, pyoluteorin, and 2,4-diacetylphloroglucinol.

### *P. piscis* 75 encodes a robust resistome

Although one *Xenorhabdus* species may be the natural endosymbiont of several *Steinernema* species, the reverse is not true: one *Steinernema* species naturally hosts one *Xenorhabdus* species only [69]. Hence, *Steinernema* sp. 75, with which *P. piscis* 75 was associated, most probably hosts *X. griffiniae* since this was the *Xenorhabdus* endosymbiont of the conspecific *Steinernema* sp. Kalro. We hypothesised that for *P. piscis* 75 to be the predominant cultivable bacterium in the haemocoel of *Steinernema* sp. 75-infected *G. mellonella* cadavers, it encodes a diverse resistome. This resistome would confer resistance against any *X. griffiniae* antimicrobial that is secreted into the *G. mellonella* haemocoel during nematode infection. To test this hypothesis, we first identified genes in the *P. piscis* 75 genome, which were predicted to encode resistance. In total, they were 39 and were predicted to encode resistance to the following antimicrobials: triclosan, phenicols, cationic antimicrobial peptides, fosfomycin, flouroquinolones, macrolides, aminoglycosides, benzalkonium chloride, aminocoumarin, diaminopyrimidines, nitrofurans, acridine dyes, cephamycin, sulfonamide, tetracyclines including glycylcycline, and betalactams including carbapenem, cephalosporin, monobactams and penams (Supplementary workbook 1).

**Fig. 4.**
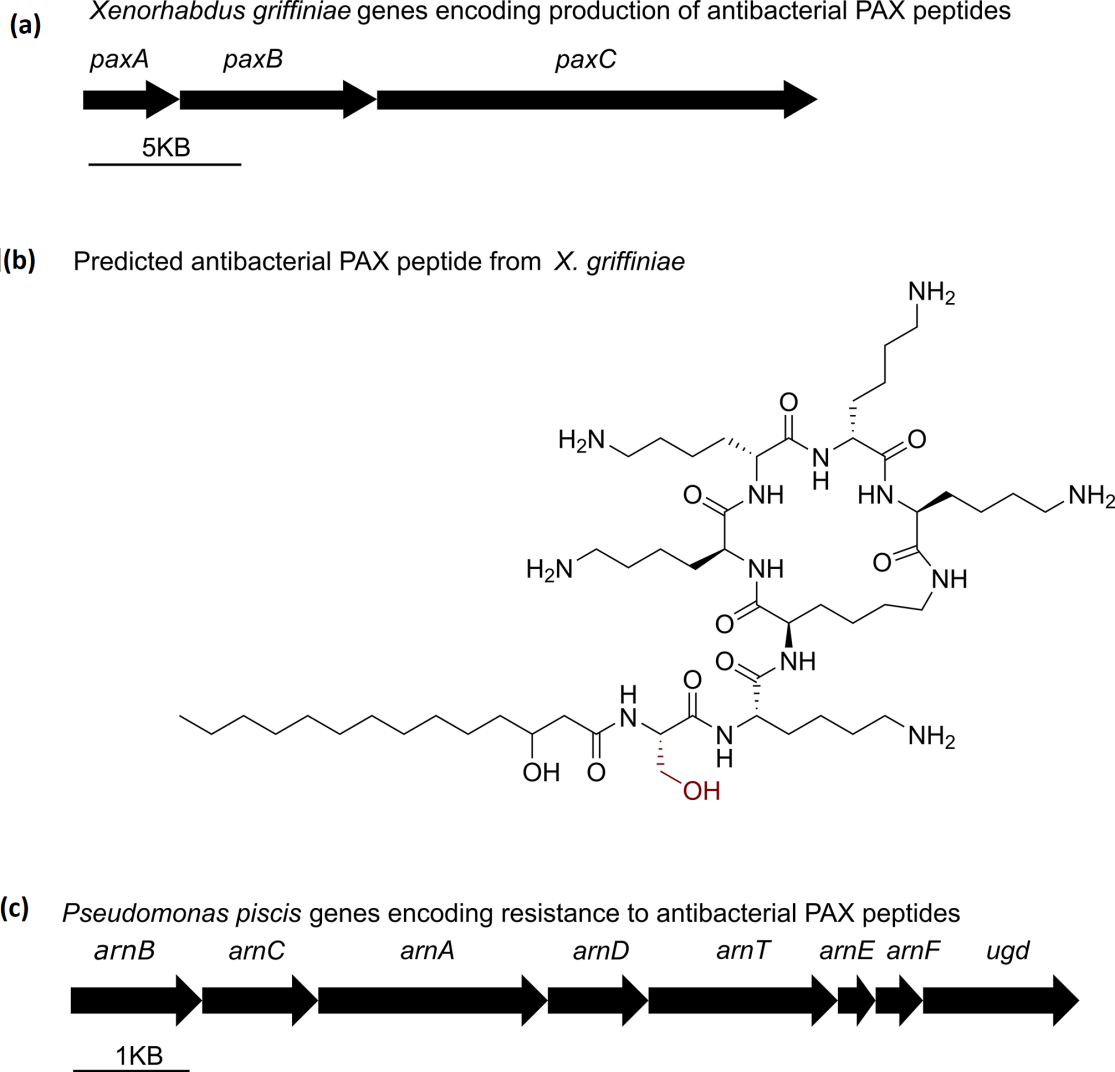
Prediction of genes encoding production of an antibacterial by *Xenorhabdus griffiniae* and genes encoding resistance to this antibacterial by *Pseudomonas piscis* 75. (**a**) The *paxABC* biosynthetic gene cluster (BGC) is predicted to encode the biosynthesis PAX lipopeptides, which are novel derivatives due to the building block in red. (**c**) The *arnBCADTEF-ugd* operon of *P. piscis* 75 is predicted to encode resistance to PAX peptides.

On the other hand, in the *X. griffiniae* Kalro genome, the putative *Xenorhabdus* endosymbiont of *Steinernema* sp. 75, we identified *paxABC* [70] as the only known BGC that encodes the production of an antibacterial, i.e. the PAX lipopeptides [71]. The cationic nature of PAX lipopeptides is due to the six lysine monomers in its heptapeptide backbone (Fig. 4). Notably, the predicted *X. griffiniae* PAX lipopeptide differed from those of *X. nematophila* F1 [71], *X.nematophila* HGB081 [70], *X. doucetiae* ^T^ [72], and *X. khoisanae* SB10 [73] by having ^L^Ser at position one instead of Gly (Fig. 4), since its corresponding Stachelhaus code was DVWHLSLIDK and not DILQIGLIWK. In terms of mechanism of action, the cationic PAX lipopeptides were shown to bind to negatively charged bacterial membranes and hence suggested to cause membrane rupture and eventual death of competing bacteria within the haemocoel of *Steinernema*-infected insect cadavers [72].

Since *P. piscis* 75 was isolated from the haemocoel of *Steinernema* sp. 75-infected cadavers, we identified genes that likely encoded resistance against PAX lipopeptides. *P. piscis* 75 contained the *arnBCADTEF-ugd* operon (Fig. 4), which upon mutation or alteration in its regulation, results in resistance to the cationic non-ribosomal antimicrobial lipopeptide polymyxin. This is due to 4-amino-4-deoxy-l-arabinose (l-Ara4N)-mediated modification of Lipid A, which creates positively charged lipopolysaccharides, which make the outer membrane repulsive to cationic lipopeptides [74]. The *arnBCADTEF-ugd* operon is regulated by products of *pmrAB* genes, and specific mutations in these genes cause over-expression of the operon that then results in polymyxin resistance [74]. Our comparison of amino acid sequences encoded by *pmrB* genes of strain 75 (locus tag: QL104_06755) and *P. aeruginosa* ^T^ (locus tag: PA4777) did not identify any of the specific mutations associated with polymyxin resistance [75]. Nonetheless, the presence of the *arnBCADTEF-ugd* operon in the *P. piscis* 75 genome demonstrates its potential to encode resistance to PAX peptides. Although PAX lipopeptides are likely not the only antibacterial compounds *X. griffiniae* produces – *Xenorhabdus* bacteria often produce a diverse array of antimicrobials [76] – the robust resistome encoded by *P. piscis* 75 is a potential key contributor to its prevalence in the haemocoel of the *Steinernema* sp. 75-infected *G. mellonella* cadavers several days after infection. Our findings may explain why *P. chlororaphis* PCLRT03 was shown to dominate *S. feltiae*-infected cadavers at the expense of the nematodes’ *Xenorhabdus* endosymbionts, seven days post-infection [77].

## Conclusion

We isolated strains of *Steinernema*-associated *X. griffiniae* and *P. piscis* from the haemocoel of *Steinernema*-infected cadavers. Both *X. griffiniae* strains Kalro and 97 belonged to the same subspecies as previously characterised *X. griffiniae* strains XN45 and VH1. *P. piscis* 75 phylogenetically clustered with highly insecticidal pseudomonads. The *P. piscis* 75 genome encoded the production of the insect toxins rhizoxin and orfamides, and the antimicrobials pyrrolnitrin, pyocyanin, pyoluteorin, and 2,4-diacetylphloroglucinol. Moreover, the *P. piscis* 75 genome had 39 genes that were predicted to confer resistance to over ten classes of antibiotics. *P. piscis* 75 hence has the potential to produce insect toxins and dominate EPN-infected cadavers through resisting antimicrobials produced by competing bacterial colonisers.

This study generated novel insights into the microbiology of EPNs: *Steinermema*-associated *P. piscis* bacteria can produce insect toxins, which are likely to contribute to host nematode entomopathogenicity. Such insights are applicable in the selection of strains for dual biological control agents (BCA) that combine entomopathogenic pseudomonads (EPP) with EPNs [78]. *P. piscis* 75 is a good candidate for EPP +EPN BCAs, as it is unlikely to be incompatible with *Steinernema* EPNs.

The complete genome sequences from this study provide the missing data for deep dives into both *X. griffiniae* and *P. piscis* pangenomes. Pangenome studies on these two species have been hampered by their few number of publicly available genomes [19, 55]. Lastly, this study has revealed *P. piscis* 75 BGCs. Some of these are predicted to encode the biosynthesis of molecules whose structures remain unknown. Refactoring these BGCs can aid the structure elucidation of the molecules, whose biosynthesis the BGCs encode. In this regard, refactoring the *P. piscis* 75 *ofaA-C* BGC would accelerate the confirmation of the chemical structures we predicted for the novel orfamide B derivatives. Similarly, refactoring unknown *P. piscis* 75 BGCs can lead to the isolation of considerable quantities of the target compounds. This in turn aids both their structure and bioactivity elucidation. In an age plagued with exacerbating pesticide and antimicrobial resistance, determining the structures and functions of novel natural products is of increasing importance.

## Supplementary Data

Supplementary material 1

Supplementary material 2
