# Supplementary material for: The genome of a steinernematid-associated Pseudomonas piscis bacterium encodes the biosynthesis of insect toxins
Source: Access Microbiol. 2023 Oct 5;5(10):000659.v3. doi: 10.1099/acmi.0.000659.v3 (PMC10634486; doi:10.1099/acmi.0.000659.v3)
Supplement: Supplementary material 1 [file acmi-5-659.v3-s001.pdf]

The genome of a steinernematid-associated *Pseudomonas piscis* bacterium encodes the biosynthesis of insect toxins

Ryan M. Awori[1,2], Prasad Hendre[1], Nelson O. Amugune[3]

[1]International Centre for Research on Agroforestry, P. O. Box 30677-00100, Nairobi, Kenya

[2]Elakistos Biosciences, P. O. Box 19301, Nairobi Kenya

[3]Department of Biology, University of Nairobi, P. O. Box 30197-00100 Nairobi, Kenya

Author for correspondence: Ryan Musumba Awori, Elakistos Biosciences, P. O. Box 19301-00100, Nairobi, Kenya, ryan-musumba.awori@elakistosbiosciences.com

Supplementary Information

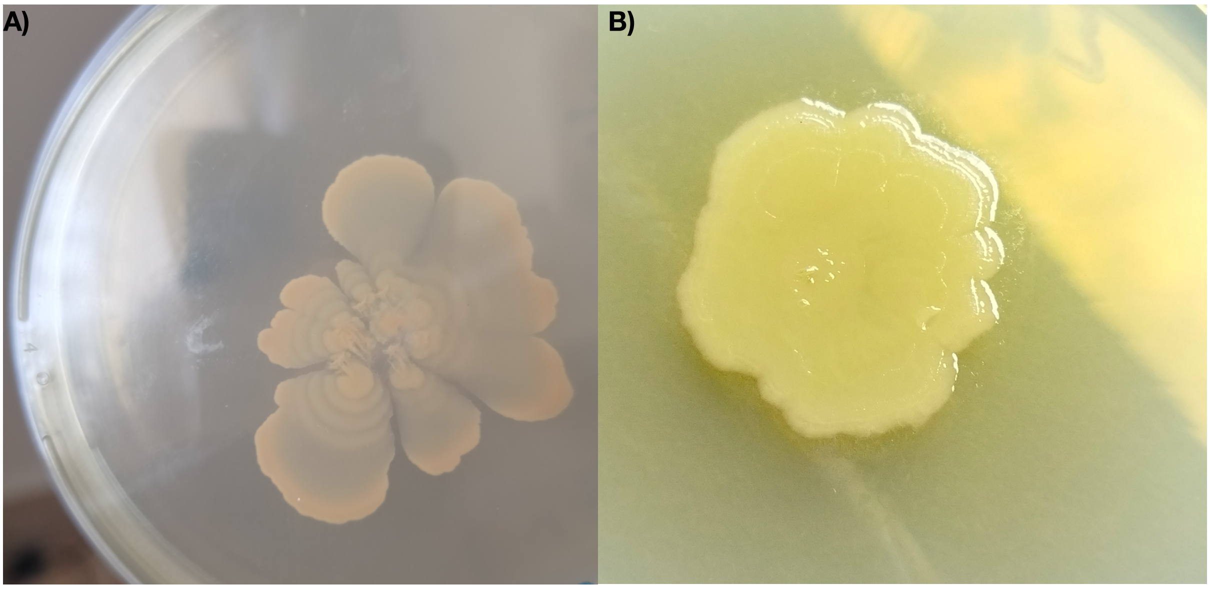

Figure S1. Photographs of colonies of *Steinernema*-associated bacteria. Colonies of A) *Xenorhabdus* griffiniae Kalro which was isolated from *Steinernema* sp. Kalro. Colonies of B) *Pseudomonas piscis* 75 which was isolated from *Steinernema* sp. 75. Both colonies displayed swarming patterns. The colony morphology for *X. griffiniae* Kalro was similar to that of *X. griffiniae* 97, which was isolated from *Steinernema* sp. 97.
